# Author Correction: Metabolite signatures of diverse *Camellia sinensis* tea populations

**DOI:** 10.1038/s41467-021-22150-y

**Published:** 2021-03-17

**Authors:** Xiaomin Yu, Jiajing Xiao, Si Chen, Yuan Yu, Jianqiang Ma, Yuzhen Lin, Ruizi Li, Jun Lin, Zhijun Fu, Qiongqiong Zhou, Qianlin Chao, Liang Chen, Zhenbiao Yang, Renyi Liu

**Affiliations:** 1 FAFU-UCR Joint Center for Horticultural Biology and Metabolomics, Haixia Institute of Science and Technology, Fujian Agriculture and Forestry University, 350002 Fuzhou, China; 2 Shanghai Center for Plant Stress Biology, Chinese Academy of Sciences, 3888 Chenhua Road, 201602 Shanghai, China; 3 University of Chinese Academy of Sciences, 100049 Beijing, China; 4 Key Laboratory of Tea Biology and Resources Utilization, Ministry of Agriculture and Rural Affairs, Tea Research Institute, Chinese Academy of Agricultural Sciences, 310008 Hangzhou, China; 5 College of Horticulture, Henan Agricultural University, 450000 Zhengzhou, China; 6 Wuyi Star Tea Industry Co., Ltd, 354300 Wuyishan, China; 7 Institute of Integrative Genome Biology, University of California at Riverside, Riverside, CA 92521 USA; 8 Department of Botany and Plant Sciences, University of California at Riverside, Riverside, CA 92521 USA; 9 Center for Agroforestry Mega Data Science, Haixia Institute of Science and Technology, Fujian Agriculture and Forestry University, 350002 Fuzhou, China

**Keywords:** Metabolomics, Genome informatics, Natural variation in plants, Plant evolution, Secondary metabolism

Correction to: *Nature Communications* 10.1038/s41467-020-19441-1, published online 04 November 2020.

The original version of this Article contained an error in the Acknowledgements, which incorrectly read ‘This work was supported by the Fujian Agriculture and Forestry University (FAFU) Construction Project for Technological Innovation and Service System of Tea Industry Chain (K1520005A02) and other funds from FAFU to X.Y., Z.Y. and R.L.’ The correct version states ‘Y.Y.’ in place of ‘Z.Y.’. This has been corrected in both the PDF and HTML versions of the Article.

