# Supplementary material for: Metabolite signatures of diverse Camellia sinensis tea populations
Source: Nat Commun. 2020 Nov 4;11:5586. doi: 10.1038/s41467-020-19441-1 (PMC7642434; doi:10.1038/s41467-020-19441-1)
Supplement: Supplementary file 1 — Supplementary Information [file 41467_2020_19441_MOESM1_ESM.pdf]

Metabolite signatures of diverse *Camellia sinensis* tea populations

Yu *et al*.

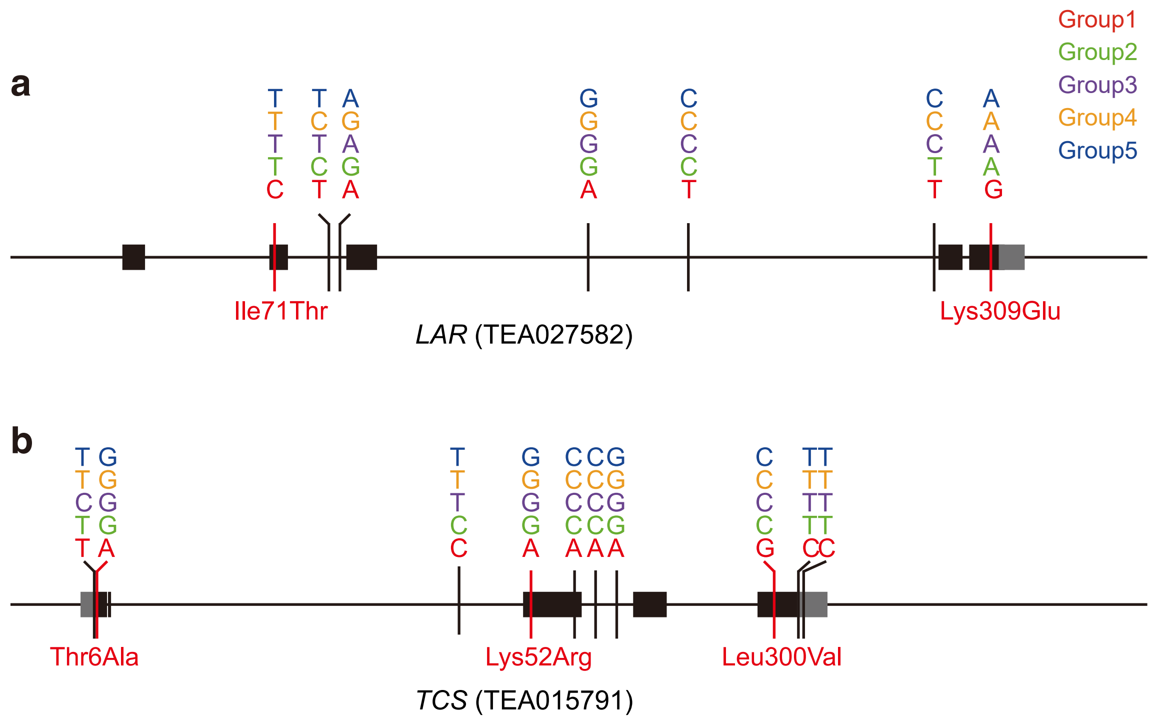

Supplementary Figure 1. Examples of signature SNPs that separate different tea groups. a The *LAR* gene (TEA027582), which encodes a leucocyanidin reductase and is involved in catechin biosynthesis, contains 7 SNPs on which major alleles are different among tea accessions. **b** The *TCS* gene (TEA015791), which encodes a caffeine synthase and is involved in caffeine biosynthesis, contains 10 SNPs on which major alleles are different between CSS and CSA groups. Nonsynonymous SNPs were plotted with red lines.

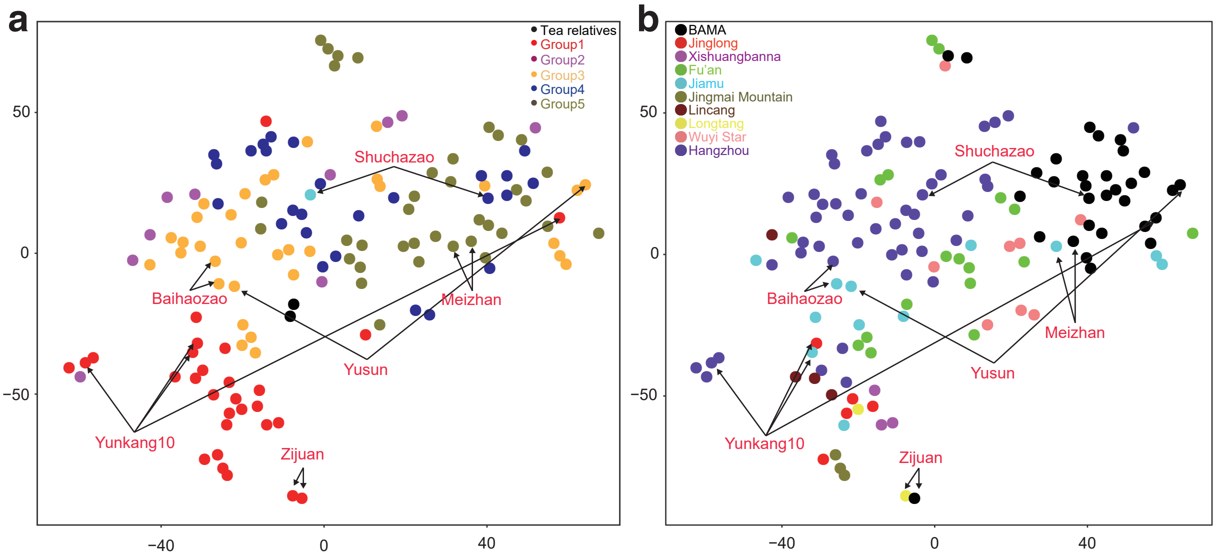

Supplementary Figure 2. Global clustering analysis of metabolite profiles using t-SNE. a Metabolite concentration clustering with each tea accession represented by a dot in different colors according to its phylogenetic group. The tea accessions in group 1 tend to cluster together (indicated by a black circle). **b** Metabolite clustering with each tea accession represented by a dot in different colors according to its growing location.

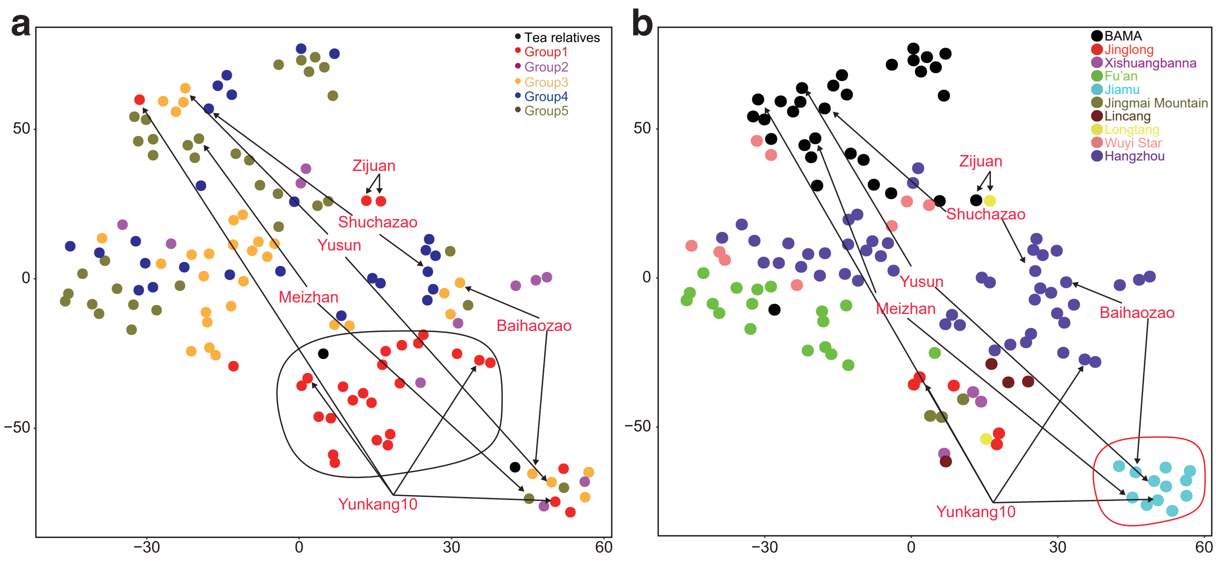

Supplementary Figure 3. Global clustering analysis of gene expression profiles using t-SNE. a Gene expression clustering with each tea sample represented by a dot in different colors according to its phylogenetic group. The tea accessions in group 1 tend to cluster together (indicated by a black circle). **b** Gene expression clustering with each tea sample represented by a dot in different colors according to its growing location. Tea accessions grown in a tea plantation with high elevation in Yunnan tend to cluster together regardless the genotype (indicated by a red circle).

Supplementary Table 1. List of tea accessions from which second-leaf samples were collected and analyzed in this study

Sample ID	Accession name	Geographical origin	Sample site
S1	Fuyun 6	Fujian	Tea Research Institute, Fujian Academy of Agricultural Science, Fu'an, Fujian
S2	Fuyun 7	Fujian	Tea Research Institute, Fujian Academy of Agricultural Science, Fu'an, Fujian
S4	Mingke 2	Fujian	Tea Research Institute, Fujian Academy of Agricultural Science, Fu'an, Fujian
S5	Yuemingxiang	Fujian	Tea Research Institute, Fujian Academy of Agricultural Science, Fu'an, Fujian
S6	Mingke 1	Fujian	Tea Research Institute, Fujian Academy of Agricultural Science, Fu'an, Fujian
S7	Huangqi	Fujian	Tea Research Institute, Fujian Academy of Agricultural Science, Fu'an, Fujian
S8	Fuyun 595	Fujian	Tea Research Institute, Fujian Academy of Agricultural Science, Fu'an, Fujian
S9	Chaoyang	Fujian	Tea Research Institute, Fujian Academy of Agricultural Science, Fu'an, Fujian
S10	Dangui	Fujian	Tea Research Institute, Fujian Academy of Agricultural Science, Fu'an, Fujian
S12	Chunlan	Fujian	Tea Research Institute, Fujian Academy of Agricultural Science, Fu'an, Fujian
S13	Zaochunhao	Fujian	Tea Research Institute, Fujian Academy of Agricultural Science, Fu'an, Fujian
S14	Jinmudan	Fujian	Tea Research Institute, Fujian Academy of Agricultural Science, Fu'an, Fujian
S20	Fuding Dabaicha	Fujian	Tea Research Institute, Fujian Academy of Agricultural Science, Fu'an, Fujian
S21	Fuding Dahaocha	Fujian	Tea Research Institute, Fujian Academy of Agricultural Science, Fu'an, Fujian
S22	Fu’an Dabaicha	Fujian	Tea Research Institute, Fujian Academy of Agricultural Science, Fu'an, Fujian
S36	Jiulong Dabaicha	Fujian	Tea Research Institute, Fujian Academy of Agricultural Science, Fu'an, Fujian
S38	Xingrencha	Fujian	Tea Research Institute, Fujian Academy of Agricultural Science, Fu'an, Fujian
S40	Xiapu Chunbolv	Fujian	Tea Research Institute, Fujian Academy of Agricultural Science, Fu'an, Fujian
S41	Zijuan	Yunnan	BAMA Tea Industry Co., Ltd., Anxi, Fujian
S42	Jiulongpao	Fujian	BAMA Tea Industry Co., Ltd., Anxi, Fujian
S43	Benshan	Fujian	BAMA Tea Industry Co., Ltd., Anxi, Fujian
S44	Tieguanyin	Fujian	BAMA Tea Industry Co., Ltd., Anxi, Fujian
S45	Meizhan	Fujian	BAMA Tea Industry Co., Ltd., Anxi, Fujian
S46	Beidong	Fujian	BAMA Tea Industry Co., Ltd., Anxi, Fujian
S47	Huangdan	Fujian	BAMA Tea Industry Co., Ltd., Anxi, Fujian
S48	Aijiao Wulong	Fujian	BAMA Tea Industry Co., Ltd., Anxi, Fujian

S49	Tieluohan	Fujian	BAMA Tea Industry Co., Ltd., Anxi, Fujian
S50	Jinsuochi	Fujian	BAMA Tea Industry Co., Ltd., Anxi, Fujian
S51	Taoxingye	Yunnan	BAMA Tea Industry Co., Ltd., Anxi, Fujian
S52	Baxian	Fujian	BAMA Tea Industry Co., Ltd., Anxi, Fujian
S53	Baiye 1	Zhejiang	BAMA Tea Industry Co., Ltd., Anxi, Fujian
S54	Huangjinya	Zhejiang	BAMA Tea Industry Co., Ltd., Anxi, Fujian
S55	Yunkang 10	Yunnan	BAMA Tea Industry Co., Ltd., Anxi, Fujian
S56	Yulv	Hunan	BAMA Tea Industry Co., Ltd., Anxi, Fujian
S57	Undetermined 1	Zhejiang	BAMA Tea Industry Co., Ltd., Anxi, Fujian
S58	Shuchazao	Anhui	BAMA Tea Industry Co., Ltd., Anxi, Fujian
S59	Jiaming 1	Zhejiang	BAMA Tea Industry Co., Ltd., Anxi, Fujian
S60	Qiannianxue	Zhejiang	BAMA Tea Industry Co., Ltd., Anxi, Fujian
S61	Yusun	Hunan	BAMA Tea Industry Co., Ltd., Anxi, Fujian
S62	Xueya 100	Yunnan	BAMA Tea Industry Co., Ltd., Anxi, Fujian
S63	Maoxie	Fujian	BAMA Tea Industry Co., Ltd., Anxi, Fujian
S64	Taicha 12	Taiwan	BAMA Tea Industry Co., Ltd., Anxi, Fujian
S65	Shuixian	Fujian	BAMA Tea Industry Co., Ltd., Anxi, Fujian
S66	Qilan	Fujian	BAMA Tea Industry Co., Ltd., Anxi, Fujian
S67	Ruixiang	Fujian	BAMA Tea Industry Co., Ltd., Anxi, Fujian
S68	Chuntaoxiang	Fujian	BAMA Tea Industry Co., Ltd., Anxi, Fujian
S69	Duanjie Baihao	Yunnan	BAMA Tea Industry Co., Ltd., Anxi, Fujian
S70	Aifeng	Yunnan	BAMA Tea Industry Co., Ltd., Anxi, Fujian
S71	Yungui	Yunnan	BAMA Tea Industry Co., Ltd., Anxi, Fujian
S73	Jianbohuang 13	Hunan	China National Germplasm Hangzhou Tea Repository, Tea Research Institute, Chinese Academy of Agricultural Science, Hangzhou Zhejiang
S74	Shangmeizhou	Jiangxi	China National Germplasm Hangzhou Tea Repository, Tea Research Institute, Chinese Academy of Agricultural Science, Hangzhou Zhejiang
S75	Zhuyeqi 12	Hunan	China National Germplasm Hangzhou Tea Repository, Tea Research Institute, Chinese Academy of Agricultural Science, Hangzhou Zhejiang
S76	Ningzhou 2	Jiangxi	China National Germplasm Hangzhou Tea Repository, Tea Research Institute, Chinese Academy of Agricultural Science, Hangzhou Zhejiang
S77	Zhenghe Dabaicha	Fujian	China National Germplasm Hangzhou Tea Repository, Tea Research Institute, Chinese Academy of Agricultural Science, Hangzhou Zhejiang
S78	Shuchazao	Anhui	China National Germplasm Hangzhou Tea Repository, Tea Research Institute, Chinese Academy of Agricultural Science, Hangzhou Zhejiang

S79	Damianbai	Jiangxi	China National Germplasm Hangzhou Tea Repository, Tea Research Institute, Chinese Academy of Agricultural Science, Hangzhou Zhejiang
S80	Xicha 5	Jiangsu	China National Germplasm Hangzhou Tea Repository, Tea Research Institute, Chinese Academy of Agricultural Science, Hangzhou Zhejiang
S81	Xicha 11	Jiangsu	China National Germplasm Hangzhou Tea Repository, Tea Research Institute, Chinese Academy of Agricultural Science, Hangzhou Zhejiang
S82	Zhuyeqi 9	Hunan	China National Germplasm Hangzhou Tea Repository, Tea Research Institute, Chinese Academy of Agricultural Science, Hangzhou Zhejiang
S83	Baihaozao	Hunan	China National Germplasm Hangzhou Tea Repository, Tea Research Institute, Chinese Academy of Agricultural Science, Hangzhou Zhejiang
S84	Fuzao 2	Anhui	China National Germplasm Hangzhou Tea Repository, Tea Research Institute, Chinese Academy of Agricultural Science, Hangzhou Zhejiang
S85	Anhui 7	Anhui	China National Germplasm Hangzhou Tea Repository, Tea Research Institute, Chinese Academy of Agricultural Science, Hangzhou Zhejiang
S86	Echa1	Hubei	China National Germplasm Hangzhou Tea Repository, Tea Research Institute, Chinese Academy of Agricultural Science, Hangzhou Zhejiang
S87	Yangshulin 783	Anhui	China National Germplasm Hangzhou Tea Repository, Tea Research Institute, Chinese Academy of Agricultural Science, Hangzhou Zhejiang
S88	Wannong 95	Anhui	China National Germplasm Hangzhou Tea Repository, Tea Research Institute, Chinese Academy of Agricultural Science, Hangzhou Zhejiang
S89	Qingfeng	Zhejiang	China National Germplasm Hangzhou Tea Repository, Tea Research Institute, Chinese Academy of Agricultural Science, Hangzhou Zhejiang
S90	Jinfeng	Zhejiang	China National Germplasm Hangzhou Tea Repository, Tea Research Institute, Chinese Academy of Agricultural Science, Hangzhou Zhejiang
S91	Echa 4	Hubei	China National Germplasm Hangzhou Tea Repository, Tea Research Institute, Chinese Academy of Agricultural Science, Hangzhou Zhejiang
S92	Guilv1	Guangxi	China National Germplasm Hangzhou Tea Repository, Tea Research Institute, Chinese Academy of Agricultural Science, Hangzhou Zhejiang
S93	Maolv	Zhejiang	China National Germplasm Hangzhou Tea Repository, Tea Research Institute, Chinese Academy of Agricultural Science, Hangzhou Zhejiang
S94	Zhongcha 102	Zhejiang	China National Germplasm Hangzhou Tea Repository, Tea Research Institute, Chinese Academy of Agricultural Science, Hangzhou Zhejiang
S95	Juhuachun	Zhejiang	China National Germplasm Hangzhou Tea Repository, Tea Research Institute, Chinese Academy of Agricultural Science, Hangzhou Zhejiang
S96	Zhenong 113	Zhejiang	China National Germplasm Hangzhou Tea Repository, Tea Research Institute, Chinese Academy of Agricultural Science, Hangzhou Zhejiang

S97	Hanlv	Zhejiang	China National Germplasm Hangzhou Tea Repository, Tea Research Institute, Chinese Academy of Agricultural Science, Hangzhou Zhejiang
S98	Zhenong 12	Zhejiang	China National Germplasm Hangzhou Tea Repository, Tea Research Institute, Chinese Academy of Agricultural Science, Hangzhou Zhejiang
S99	Zhenong 21	Zhejiang	China National Germplasm Hangzhou Tea Repository, Tea Research Institute, Chinese Academy of Agricultural Science, Hangzhou Zhejiang
S100	Baimao 2	Guangdong	China National Germplasm Hangzhou Tea Repository, Tea Research Institute, Chinese Academy of Agricultural Science, Hangzhou Zhejiang
S101	Zhongcha 302	Zhejiang	China National Germplasm Hangzhou Tea Repository, Tea Research Institute, Chinese Academy of Agricultural Science, Hangzhou Zhejiang
S102	Zhongcha 108	Zhejiang	China National Germplasm Hangzhou Tea Repository, Tea Research Institute, Chinese Academy of Agricultural Science, Hangzhou Zhejiang
S103	Shifocui	Anhui	China National Germplasm Hangzhou Tea Repository, Tea Research Institute, Chinese Academy of Agricultural Science, Hangzhou Zhejiang
S104	Shuyong 906	Chongqing	China National Germplasm Hangzhou Tea Repository, Tea Research Institute, Chinese Academy of Agricultural Science, Hangzhou Zhejiang
S105	Shuyong 401	Chongqing	China National Germplasm Hangzhou Tea Repository, Tea Research Institute, Chinese Academy of Agricultural Science, Hangzhou Zhejiang
S106	Qiancha 8	Guizhou	China National Germplasm Hangzhou Tea Repository, Tea Research Institute, Chinese Academy of Agricultural Science, Hangzhou Zhejiang
S107	Qianmei 419	Guizhou	China National Germplasm Hangzhou Tea Repository, Tea Research Institute, Chinese Academy of Agricultural Science, Hangzhou Zhejiang
S108	Tianfucha 28	Sichuan	China National Germplasm Hangzhou Tea Repository, Tea Research Institute, Chinese Academy of Agricultural Science, Hangzhou Zhejiang
S109	Wancha 91	Anhui	China National Germplasm Hangzhou Tea Repository, Tea Research Institute, Chinese Academy of Agricultural Science, Hangzhou Zhejiang
S110	Huangmeigui	Fujian	China National Germplasm Hangzhou Tea Repository, Tea Research Institute, Chinese Academy of Agricultural Science, Hangzhou Zhejiang
S111	Shuyong 1	Chongqing	China National Germplasm Hangzhou Tea Repository, Tea Research Institute, Chinese Academy of Agricultural Science, Hangzhou Zhejiang
S112	Nanjiang 1	Chongqing	China National Germplasm Hangzhou Tea Repository, Tea Research Institute, Chinese Academy of Agricultural Science, Hangzhou Zhejiang
S113	Guihong 3	Gunagxi	China National Germplasm Hangzhou Tea Repository, Tea Research Institute, Chinese Academy of Agricultural Science, Hangzhou Zhejiang
S114	Yunkang 10	Yunnan	China National Germplasm Hangzhou Tea Repository, Tea Research Institute, Chinese Academy of Agricultural Science, Hangzhou Zhejiang

S115	Lingtou Dancong	Guangdong	China National Germplasm Hangzhou Tea Repository, Tea Research Institute, Chinese Academy of Agricultural Science, Hangzhou Zhejiang
S116	Yinghong 1	Guangdong	China National Germplasm Hangzhou Tea Repository, Tea Research Institute, Chinese Academy of Agricultural Science, Hangzhou Zhejiang
S117	Zhenong 117	Zhejiang	China National Germplasm Hangzhou Tea Repository, Tea Research Institute, Chinese Academy of Agricultural Science, Hangzhou Zhejiang
S118	Longjing 43	Zhejiang	China National Germplasm Hangzhou Tea Repository, Tea Research Institute, Chinese Academy of Agricultural Science, Hangzhou Zhejiang
S119	Mingshan Baihao 131	Sichuan	China National Germplasm Hangzhou Tea Repository, Tea Research Institute, Chinese Academy of Agricultural Science, Hangzhou Zhejiang
S120	Hongyan 7	Guangdong	China National Germplasm Hangzhou Tea Repository, Tea Research Institute, Chinese Academy of Agricultural Science, Hangzhou Zhejiang
S121	Zaobaijian 5	Chongqing	China National Germplasm Hangzhou Tea Repository, Tea Research Institute, Chinese Academy of Agricultural Science, Hangzhou Zhejiang
S122	Changye Baihao	Yunnan	Longyuan Tea Industry Co., Ltd., Dadugang, Jinghong, Yunnan
S123	Yunkang 10	Yunnan	Longyuan Tea Industry Co., Ltd., Dadugang, Jinghong, Yunnan
S124	Dadonggang Dayecha	Yunnan	Longyuan Tea Industry Co., Ltd., Dadugang, Jinghong, Yunnan
S126	Jinggu Dabaicha	Yunnan	Longyuan Tea Industry Co., Ltd., Dadugang, Jinghong, Yunnan
S127	Ziya	Yunnan	Longyuan Tea Industry Co., Ltd., Dadugang, Jinghong, Yunnan
S129	Nannuoshan Dayecha 2	Yunnan	Nannuo Mountain, Xishuangbanna, Yunnan
S131	Nannuoshan Dayecha 3	Yunnan	Nannuo Mountain, Xishuangbanna, Yunnan
S132	Nannuoshan Zhongxiaoyecha	Yunnan	Nannuo Mountain, Xishuangbanna, Yunnan
S133	Rougui	Fujian	Wuyi Star Tea Industry Co., Ltd., Wuyishan City, Fujian
S134	Dahongpao	Fujian	Wuyi Star Tea Industry Co., Ltd., Wuyishan City, Fujian
S135	Baijiguan	Fujian	Wuyi Star Tea Industry Co., Ltd., Wuyishan City, Fujian
S136	Queshe	Fujian	Wuyi Star Tea Industry Co., Ltd., Wuyishan City, Fujian
S137	Shuijingui	Fujian	Wuyi Star Tea Industry Co., Ltd., Wuyishan City, Fujian
S138	Bantianyao	Fujian	Wuyi Star Tea Industry Co., Ltd., Wuyishan City, Fujian
S140	Guazijin	Fujian	Wuyi Star Tea Industry Co., Ltd., Wuyishan City, Fujian
S141	Foshou	Fujian	Wuyi Star Tea Industry Co., Ltd., Wuyishan City, Fujian
S142	Xiaoye Maoxie	Fujian	Wuyi Star Tea Industry Co., Ltd., Wuyishan City, Fujian
S143	Jingmaishan Dayecha 1	Yunnan	Jingmai Mountain, Lancang, Puer, Yunnan

S144	Jingmaishan Dayecha 2	Yunnan	Jingmai Mountain, Lancang, Puer, Yunnan
S145	Jingmaishan Dayecha 3	Yunnan	Jingmai Mountain, Lancang, Puer, Yunnan
S146	Undetermined 2	Yunnan	Menglang, Lancang, Puer, Yunnan
S147	Zijuan	Yunnan	Menglang, Lancang, Puer, Yunnan
S148	Xianggui Yinhao	Yunnan	Lincang Tea Research Institute, Lincang, Yunnan
S149	Xianggui Chunzao	Yunnan	Lincang Tea Research Institute, Lincang, Yunnan
S150	Shilixiang	Yunnan	Lincang Tea Research Institute, Lincang, Yunnan
S151	Qingshui 3	Yunnan	Lincang Tea Research Institute, Lincang, Yunnan
S152	Lincang Dayecha	Yunnan	Jiamu Tea Industry Co., Ltd., Yunxian, Lincang, Yunnan
S153	Bixiangzao	Hunan	Jiamu Tea Industry Co., Ltd., Yunxian, Lincang, Yunnan
S154	Zhuyeqi	Hunan	Jiamu Tea Industry Co., Ltd., Yunxian, Lincang, Yunnan
S155	Yusun	Hunan	Jiamu Tea Industry Co., Ltd., Yunxian, Lincang, Yunnan
S156	Baihaozao	Hunan	Jiamu Tea Industry Co., Ltd., Yunxian, Lincang, Yunnan
S157	Foxiang 3-3	Yunnan	Jiamu Tea Industry Co., Ltd., Yunxian, Lincang, Yunnan
S158	Wulong	Yunnan	Jiamu Tea Industry Co., Ltd., Yunxian, Lincang, Yunnan
S159	Dali Yeshengcha	Yunnan	Jiamu Tea Industry Co., Ltd., Yunxian, Lincang, Yunnan
S160	Dabaihao	Yunnan	Jiamu Tea Industry Co., Ltd., Yunxian, Lincang, Yunnan
S161	Taoyuan Daye	Hunan	Jiamu Tea Industry Co., Ltd., Yunxian, Lincang, Yunnan
S162	Meizhang	Yunnan	Jiamu Tea Industry Co., Ltd., Yunxian, Lincang, Yunnan
S164	Yunkang 10	Yunnan	Jiamu Tea Industry Co., Ltd., Yunxian, Lincang, Yunnan
