# Supplementary material for: Metabolite signatures of diverse Camellia sinensis tea populations
Source: Nat Commun. 2020 Nov 4;11:5586. doi: 10.1038/s41467-020-19441-1 (PMC7642434; doi:10.1038/s41467-020-19441-1)
Supplement: Supplementary file 3 — Description of Additional Supplementary Files [file 41467_2020_19441_MOESM3_ESM.docx]

**Description of Supplementary Files**

**File Name: Supplementary Data 1**

**Description:** Mean abundance of 752 filtered metabolic features detected by UPLC-QTOF MS under POS mode in the second leaf samples of 136 tea accessions.

**File Name: Supplementary Data 2**

**Description:** Mean abundance of 503 filtered metabolic features detected by UPLC-QTOF MS under NEG mode in the second leaf samples of 136 tea accessions.

**File Name: Supplementary Data 3**

**Description:** Details of 179 annotated metabolites detected under POS mode.

**File Name: Supplementary Data 4**

**Description:** Details of 258 annotated metabolites detected under NEG mode.

**File Name: Supplementary Data 5**

**Description:** Details of 129 differentially accumulated metabolites (DAMs) detected under POS mode.

**File Name: Supplementary Data 6**

**Description:** Details of 199 differentially accumulated metabolites (DAMs) detected under NEG mode.

**File Name: Supplementary Data 7**

**Description:** List of abbreviated and full names of the annotated metabolites included in Fig.4c.
