# Supplementary material for: Metabolite signatures of diverse Camellia sinensis tea populations
Source: Nat Commun. 2020 Nov 4;11:5586. doi: 10.1038/s41467-020-19441-1 (PMC7642434; doi:10.1038/s41467-020-19441-1)
Supplement: Supplementary file 11 — Reporting Summary [file 41467_2020_19441_MOESM11_ESM.pdf]

nature research

Corresponding author(s): Renyi Liu, Zhenbiao Yang, Liang Chen

Last updated by author(s): Oct 6, 2020

Reporting Summary

Nature Research wishes to improve the reproducibility of the work that we publish. This form provides structure for consistency and transparency in reporting. For further information on Nature Research policies, see our Editorial Policies and the Editorial Policy Checklist.

Statistics

For all statistical analyses, confirm that the following items are present in the figure legend, table legend, main text, or Methods section.

n/a	Confirmed	
☐	☒	The exact sample size (*n*) for each experimental group/condition, given as a discrete number and unit of measurement
☐	☒	A statement on whether measurements were taken from distinct samples or whether the same sample was measured repeatedly
☐	☒	The statistical test(s) used AND whether they are one- or two-sided<br>*Only common tests should be described solely by name; describe more complex techniques in the Methods section.*
☒	☐	A description of all covariates tested
☐	☒	A description of any assumptions or corrections, such as tests of normality and adjustment for multiple comparisons
☐	☒	A full description of the statistical parameters including central tendency (e.g. means) or other basic estimates (e.g. regression coefficient) AND variation (e.g. standard deviation) or associated estimates of uncertainty (e.g. confidence intervals)
☒	☐	For null hypothesis testing, the test statistic (e.g. *F*, *t*, *r*) with confidence intervals, effect sizes, degrees of freedom and *P* value noted<br>*Give P values as exact values whenever suitable.*
☒	☐	For Bayesian analysis, information on the choice of priors and Markov chain Monte Carlo settings
☒	☐	For hierarchical and complex designs, identification of the appropriate level for tests and full reporting of outcomes
☒	☐	Estimates of effect sizes (e.g. Cohen's *d*, Pearson's *r*), indicating how they were calculated

Our web collection on statistics for biologists contains articles on many of the points above.

Software and code

Policy information about availability of computer code

Data collection	MassLynx v4.1
Data analysis	Progenesis QI v2.1; SOAPnuke v2.0.1; hisat2 v2.1.0; HTseq-count v 0.11.2; t-SNE v0.15 in R version 3.5.1; EdgeR v3.28.0; GATK v4.0.4.0; ANNOVAR v20170717; FastTree v2.1.11; PLINK v1.9; STRCTURE v2.3.4; XP-CLR v1.0; Harvester v0.6.94

For manuscripts utilizing custom algorithms or software that are central to the research but not yet described in published literature, software must be made available to editors and reviewers. We strongly encourage code deposition in a community repository (e.g. GitHub). See the Nature Research guidelines for submitting code & software for further information.

Data

Policy information about availability of data

All manuscripts must include a data availability statement. This statement should provide the following information, where applicable:

- Accession codes, unique identifiers, or web links for publicly available datasets
- A list of figures that have associated raw data
- A description of any restrictions on data availability

RNA-sequencing data that support the findings of this study have been deposited to the Gene Expression Omnibus (GEO) database at the National Center for Biotechnology Information (NCBI) and are accessible with project number PRJNA562973 (https://www.ncbi.nlm.nih.gov/bioproject/PRJNA562973). Metabolomics data have been deposited to the MetaboLights database at the EMBL-European Bioinformatics Institute (EBI) with project number MTBLS1405 (https://www.ebi.ac.uk/metabolights/MTBLS1405). All other relevant data are available from the corresponding author on request. Source data are provided with this paper.

Field-specific reporting

Please select the one below that is the best fit for your research. If you are not sure, read the appropriate sections before making your selection.

- [x] Life sciences
- [ ] Behavioural & social sciences
- [ ] Ecological, evolutionary & environmental sciences

For a reference copy of the document with all sections, see nature.com/documents/nr-reporting-summary-flat.pdf

Life sciences study design

All studies must disclose on these points even when the disclosure is negative.

Sample size	136 tea accessions were collected from major tea growing areas in China. These accessions largely represent the genetic diversity of natural tea populations. For each tea accession, three biological replicates were prepared for RNA sequencing and five biological replicates were prepared for metabolomics analysis.
Data exclusions	No data were excluded for the analyses.
Replication	Three (for RNA-seq) and five (for metabolite profiling) Biological replicates for each accession were independently collected from major tea-growing regions in China from April 13th to 25th, 2018. All attempts for independent collections of biological replicates were successful.
Randomization	136 tea accessions were collected from the field in major tea growing regions. Based on their geographical origins, these accessions largely represent the diversity of tea populations in China. Randomization is not relevant for this study.
Blinding	Not applicable for this study because our aim was to dissect the genetic and metabolite diversity in major tea populations. We need to know each sample was from which accession to perform the analyses.

Reporting for specific materials, systems and methods

We require information from authors about some types of materials, experimental systems and methods used in many studies. Here, indicate whether each material, system or method listed is relevant to your study. If you are not sure if a list item applies to your research, read the appropriate section before selecting a response.

Materials & experimental systems

n/a	Involved in the study	
x		Antibodies
x		Eukaryotic cell lines
x		Palaeontology and archaeology
x		Animals and other organisms
x		Human research participants
x		Clinical data
x		Dual use research of concern

Methods

n/a	Involved in the study	
x		ChIP-seq
x		Flow cytometry
x		MRI-based neuroimaging
